# Short-term maintenance on a high-sucrose diet alleviates aging-induced sleep fragmentation in *drosophila*

**DOI:** 10.1080/19768354.2021.1997801

**Published:** 2021-11-03

**Authors:** Sang Hyuk Lee, Eun Young Kim

**Affiliations:** a Department of Biomedical Sciences, Ajou University Graduate School of Medicine, Suwon, Republic of Korea; b Department of Brain Science, Ajou University Medical Center, Suwon, Republic of Korea

**Keywords:** *Drosophila*, high-sucrose diet, aging, sleep fragmentation

## Abstract

Sleep is a fundamental behavior in an animal’s life influenced by many internal and external factors, such as aging and diet. Critically, poor sleep quality places people at risk of serious medical conditions. Because aging impairs quality of sleep, measures to improve sleep quality for elderly people are needed. Given that diet can influence many aspects of sleep, we investigated whether a high-sucrose diet (HSD) affected aging-induced sleep fragmentation using the fruit fly, *Drosophila melanogaster*. *Drosophila* is a valuable model for studying sleep due to its genetic tractability and many similarities with mammalian sleep. Total sleep duration, sleep bout numbers (SBN), and average sleep bout length (ABL) were compared between young and old flies on a normal sucrose diet (NSD) or HSD. On the NSD, old flies slept slightly more and showed increased SBN and reduced ABL, indicating increased sleep fragmentation. Short-term maintenance of flies in HSD (up to 8 days), but not long-term maintenance (up to 35 days), suppressed aging-induced sleep fragmentation. Our study provides meaningful strategies for preventing the deterioration of sleep quality in the elderly.

## Introduction

Sleep is a necessary behavior to maintain an animal’s physical and cognitive health. Poor sleep quality is associated with high risk of serious medical conditions, including obesity (Beccuti and Pannain 2011), insulin resistance and diabetes (Knutson and Van Cauter 2008), coronary heart disease (Meisinger et al. 2007; Phillips and Mannino 2007), and mental health issues (Alvaro et al. 2013; Hwang et al. 2019; Freeman et al. 2020; Jung and Noh 2021). Fragmented sleep with age adversely affects human health (Carskadon et al. 1982; Ancoli-Israel 2009). As poor quality of sleep is associated with high mortality of elderly (Wingard and Berkman 1983; Morgan et al. 1989), it is critical to develop measures to improve sleep quality.

The fruit fly, *Drosophila melanogaster*, is a valuable, highly genetically tractable model for studying animal behavior and physiology. Flies are instrumental to study sleep because of notable similarities between fly and mammalian sleep processes (Hendricks et al. 2000; Shaw et al. 2000; Cirelli 2003). Flies exhibit sustained periods of quiescence wherein they display 5 min or more with stereotyped posture and increased arousal threshold to sensory stimuli. Importantly, this rest-like behavior is reversed by sufficiently strong stimuli, which is the criterion to differentiate sleep from a coma or the effects of anesthesia. Thus, behavioral quiescence of 5 min or more is operatively defined as sleep in flies. When deprived of sleep, flies show homeostatic increases in sleep. Like humans, flies mostly sleep throughout the night and during the middle of day, known in some cultures as a *siesta*. This rhythmic sleep/wake pattern is regulated by the circadian clock (Shaw et al. 2000; Hendricks et al. 2003). Similarly to human sleep, fly sleep is regulated by a homeostatic mechanism (process S) and a circadian clock (process C) (Borbely and Wirz-Justice 1982; Daan et al. 1984; Borbely et al. 2016). In addition, brain electrical activity during sleep in flies is identifiably different from those during wake periods (Nitz et al. 2002; Bushey et al. 2015). Flies stay awake to stimulant such as caffeine or modafinil (Hendricks et al. 2000; Shaw et al. 2000; Hendricks et al. 2003) and increase sleep to hypnotics (Shaw et al. 2000).

Importantly, flies, like humans, show aging-dependent deterioration of sleep quality (Koh K et al. 2006). In aged flies, regardless of sex, the amount of daytime and nighttime sleep decrease. As the number of sleep bouts increases, the average sleep bout length shortens, a pattern indicative of fragmented sleep. With age sleep recovery after sleep deprivation declines (Vienne et al. 2016). Aging-induced sleep fragmentation may depend on many factors, such as genetic background and rearing conditions (Bushey et al. 2010).

Interestingly, Linford et al. showed that flies maintained on a high-sucrose diet (HSD) had fewer sleep bouts and increased average sleep bout length (ABL) compared to flies maintained on a low-sucrose diet (LSD) (Linford et al. 2012), suggesting that sleep is more consolidated in HSD than in LSD. Thus, we hypothesized that feeding flies HSD suppresses aging-induced sleep fragmentation. To test our hypothesis, we measured sleep of young (5 days old) and old (30 days old) flies in food with 5% sucrose (normal sucrose diet, NSD) or 20% sucrose (HSD). Short-term exposure to HSD reduced sleep fragmentation in aged flies. This effect was observed in different genetic backgrounds (i.e. *w*^1118^ and Canton-S) and regardless of sex, although the effect was diminished in females. Interestingly, short-term exposure to a high-fat diet (HFD) also reduced sleep fragmentation in aged flies. However, long-term exposure to HSD did not have a beneficial effect on aging-induced sleep deterioration. Collectively, our results suggest that controlled short-term exposure to high-nutrient food could alleviate sleep deterioration in aged flies. Further research determining the molecular mechanism through which HSD suppresses sleep fragmentation may provide valuable treatment strategies for age-related sleep issues.

## Materials and methods

### Fly strains and husbandry

*w*^1118^ isogenic and Canton-S standard laboratory strains were used in this study. Flies were raised in a standard cornmeal (5%, wt/vol), yeast (2%, wt/vol), agar (0.7%, wt/vol) and sucrose (7%, wt/vol) based food at room temperature. Adult flies were collected within 24 h of eclosion, transferred to new standard food, and aged in a 12-h light (L):12-h dark (D) cycle at 25°C. Sleep analysis was performed for 5-day-old and 30-day-old flies.

### Sleep analysis

Flies were housed individually in glass tubes containing 2% agar in addition to 5% sucrose (NSD), 20% sucrose (HSD), or 5% sucrose with 20% coconut oil (Nutiva) (HFD). Young (5-day-old) and old (30-day-old) male and female flies were used for the analysis. Flies were exposed to a 12L:12D cycle at 25°C in the incubator for 7 days of the experiment. Beam-break locomotor activity was recorded using the *Drosophila* Activity Monitoring system (Trikinetics). Sleep was defined as a period of at least 5 min of inactivity flanked by periods of activity (Hendricks et al. 2000; Shaw et al. 2000). The Counting macro (5.19.9 2016a) program was used to analyze sleep patterns and parameters (Pfeiffenberger et al. 2010).

### Immunohistochemistry and confocal imaging

Fly heads were cut open, fixed in 2% formaldehyde, and washed with 0.5% PAXD buffer (1X PBS, 5% BSA, 0.03% sodium deoxycholate, 0.03% Triton X-100). The fixed heads were dissected, and the isolated brains were permeabilized in 1% PBT for 20 min and then blocked in 0.5% PAXD containing 5% horse serum for 1 h. The following primary antibodies were added to the mixtures directly: anti-DTk antibody (Gp2) (Lee et al. 2021); anti-NC82 antibody (DSHB), diluted 1:200. The brains were washed with PAXD and incubated overnight with secondary antibodies in a blocking solution at 4°C. The following secondary antibodies were used at a 1:200 dilution: goat anti-guinea pig Alexa-488 (Thermo Fisher Scientific), goat anti-mouse Alexa-555 (Thermo Fisher Scientific). Stained brain samples were washed with PAXD, incubated in 0.1 M phosphate buffer containing 50% glycerol for 30 min, and mounted using a mounting medium. Confocal images were obtained using an LSM 800 confocal microscope (Carl Zeiss) and were processed using Zen software (ZEN Digital Imaging for Light Microscopy, Carl Zeiss). DTk intensity was determined using the ImageJ software. In the dorsal fan-shaped body (dFB), DTk fluorescence signal above the background was selected by adjusting the color threshold. The total intensity of DTk in the dFB was obtained by multiplying mean intensity and the area of the selected region.

### Statistical analysis

GraphPad Prism 8 software was used for statistical analysis. All data were observed through a D’Agostino-Pearson omnibus test for normality (*p* < 0.05). Experimental groups were compared by independent t-test (in the case of normal distribution) or Mann–Whitney *U* test (in the case of non-normal distribution). Differences were considered significant when *p < *0.05(*), *p < *0.01(**), *p < *0.001 (***), or *p < *0.0001 (****). All data represented multiple independent experiments.

## Results

### Aging-induced sleep fragmentation was reduced with a HSD

**Figure 1.**
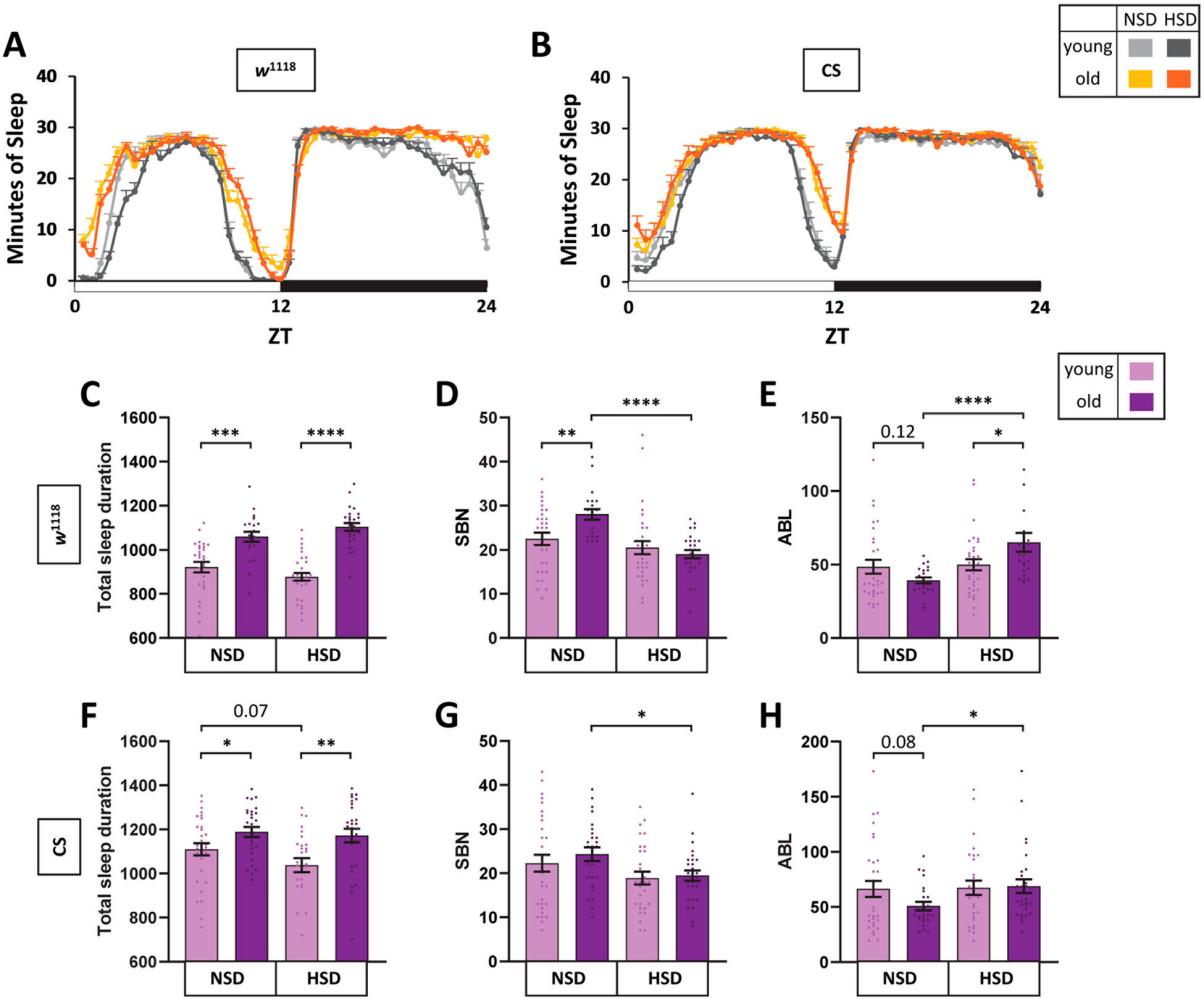
HSD reduced sleep fragmentation of old male flies. Young (5-day-old) and old (30-day-old) *w*^1118^ and Canton-S male flies were maintained in NSD or HSD in the incubator with a 12L:12D cycle at 25°and sleep was measured. (A and B) Daily sleep profiles of flies at day 7 are shown. The minutes of sleep in each 30-min bin is plotted against the Zeitgeber time (ZT). The white horizontal bar indicates light and the black horizontal bar indicates dark. (C, F) Total sleep amount, (D, G) SBN, and (E, H) ABL of individual flies were obtained. Bars represent mean ± SEM (*n* =2 1–32). Statistical significance was assessed by independent *t*-test or Mann–Whitney *U* test: *p < 0.05, **p < 0.01, ***p < 0.001, ****p < 0.0001.

To examine whether dietary sugar content affected sleep behavior depending on age, we analyzed sleep patterns of young (5 days old) and old (30 days old) isogenic *w*^1118^ male flies in food with either a normal sucrose concentration (5% sucrose, NSD) or high sucrose concentration (20% sucrose, HSD). Because sleep behavior can be affected by multiple genetic factors, we used isogenic *w*^1118^ flies for analysis. Young male flies slept in the middle of the day and throughout the night (Figure 1(A)). As reported previously (Koh K et al. 2006), old flies slept slightly more at dawn and dusk. These sleep pattern differences between young and old flies were similar for flies in both the NSD and HSD conditions.

We next analyzed sleep parameters. Old flies slept more compared to young flies in both NSD and HSD conditions (Figure 1(C)). The current paradigm of age and sleep is that total sleep amount decreases with age both in human and flies (Landolt et al. 1996; Shaw et al. 2000; Carrier et al. 2001). However, some studies have reported that total sleep amounts were either not affected by age or increased with age both in human and flies (Ohayon et al. 2004; Bushey et al. 2010; Hasan et al. 2012). Collectively, these studies suggested that the change of total sleep amount with the function of age might not be linear as we once thought. Next, we analyzed SBN and ABL. In the NSD condition, SBN was significantly increased (Figure 1(D)), and ABL was modestly reduced (Figure 1(E)) with age. On the contrary, in HSD condition, the SBN of aged flies was similar to that of young flies (Figure 1(D)). Moreover, ABL of aged flies was marginally increased compared to those of young flies in the HSD condition (Figure 1(E)). These results indicated that HSD suppressed the sleep fragmentation of old flies.

We next examined the effect of HSD on sleep fragmentation in wild-type Canton-S male flies. Differences in sleep patterns depending on age were similar but less pronounced compared to those of *w*^1118^ flies (Figure 1(B)). In the NSD condition, aged Canton-S flies showed increased sleep amount (Figure 1(F)), higher SBN (Figure 1(G)), and lower ABL (Figure 1(H)) compared to young flies. In the HSD condition, SBN and ABL were the same between young and aged flies. Together, these results indicate that HSD alleviated sleep fragmentation in Canton-S flies.

**Figure 2.**
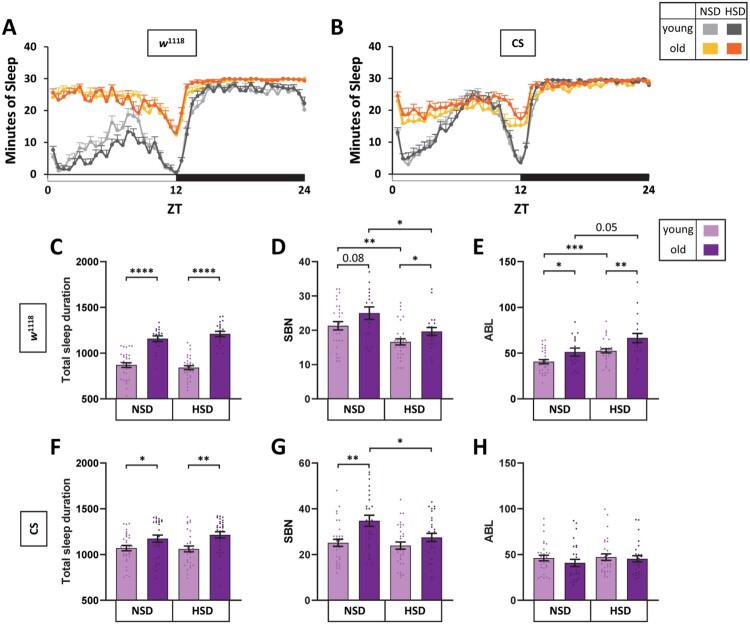
HSD reduced sleep fragmentation of old female flies was reduced. Young (5-day-old) and old (30-day-old) *w*^1118^ and Canton-S female flies were maintained in NSD or HSD in the incubator with a 12L:12D cycle at 25°and sleep was measured. (A and B) Daily sleep profiles of flies at day 7 are shown. The minutes of sleep in each 30-min bin is plotted against the ZT. The white horizontal bar indicates light and the black horizontal bar indicates dark. (C, F) Total sleep amount, (D, G) SBN, and (E, H) ABL of individual flies were obtained. Bars indicate mean ± SEM (*n* = 16–32). Statistical significance was assessed by independent *t*-test or Mann–Whitney test: *p < 0.05, **p < 0.01, ***p < 0.001, ****p < 0.0001.

The sleep patterns of male and female flies are different. Male flies slept more and manifest longer ABL especially during the daytime. Female flies show slightly higher SBN than male flies (Huber et al. 2004; Andretic and Shaw 2005; Isaac et al. 2010). Thus, we examined whether HSD reduced sleep fragmentation in old *w*^1118^ and Canton-S female flies. Sleep became evenly distributed throughout the day with age for female flies in both the NSD and HSD conditions (Figure 2(A,B)). Previous reports indicate that much older flies (e.g. 60-days-old) manifest an even distribution of sleep throughout the day both in males and females; this phenotype is present in males and even more striking in females (Koh K et al. 2006). As observed in previous studies, we found that the even distribution of sleep throughout the day as a function of age was more severe in female flies. We next analyzed sleep parameters in female flies. Similarly to male flies, old *w*^1118^ and Canton-S female flies slept more compared to young flies for both the NSD and HSD conditions (Figure 2(C,F)). In the NSD condition, SBN was increased in old *w*^1118^ and Canton S female flies, but the difference did not rise to the level of statistical significance in *w*^1118^ flies (Figure 2(D,G). ABL was increased in old *w*^1118^ flies (Figure 2(D)) but did not change in Canton-S flies (Figure 2(H)). HSD also did not change ABL in female Canton-S flies (Figure 2(D,H)). These results indicated that sleep in females may not be as fragmented as in old males. Nevertheless, the comparison between old flies in NSD versus HSD conditions indicated that SBN of HSD condition was lower and ABL of HSD condition was higher. These results indicated that, while aging-induced sleep fragmentation of female flies was not as severe as in male flies, sleep quality was enhanced by HSD. Collectively, HSD suppressed the aging-induced deterioration of sleep quality, regardless of sex and genotype.

### A HFD reduced aging-induced sleep fragmentation

**Figure 3.**
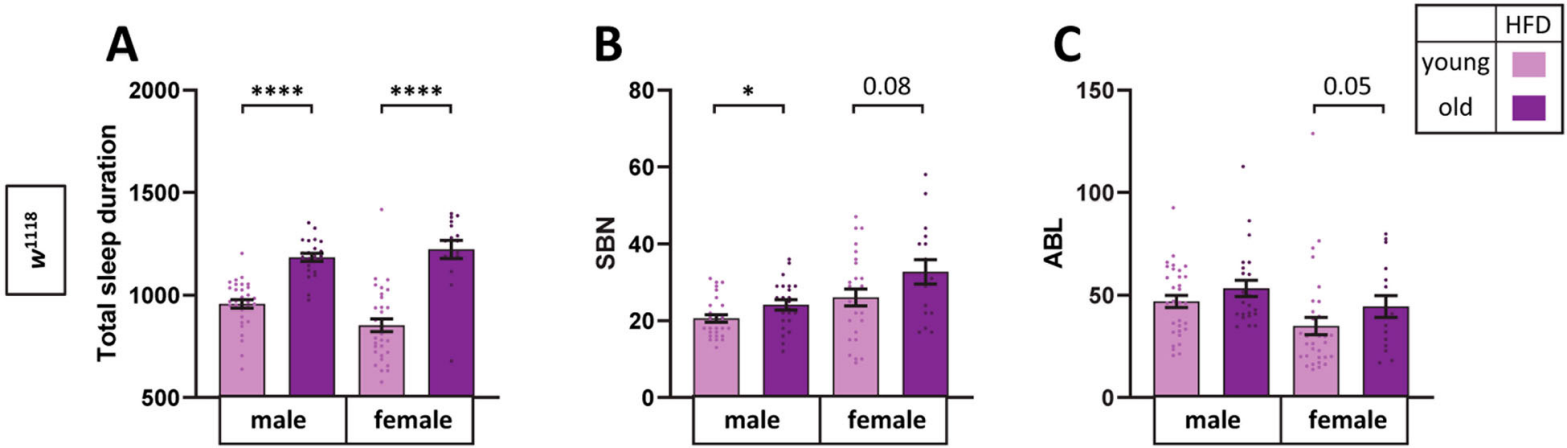
Sleep fragmentation of old female flies was reduced in HFD. Old (30-day-old) *w*^1118^ male and female flies were maintained in NSD or HFD in the incubator with a 12L:12D cycle at 25°C, and sleep was measured. (A) Total sleep amount, (B) SBN, and (C) ABL of individual flies were obtained. Bars indicate mean ± SEM (*n* = 16–32). Statistical significance was assessed by independent *t*-test or Mann–Whitney test: *p < 0.05, ****p < 0.0001.

To test whether the high-nutrient effect on aging-induced sleep fragmentation was specific to sucrose, we compared sleep parameters between young and old *w*^1118^ flies fed an HFD. In agreement with the previous figures, both male and female flies’ total sleep increased with age in HFD (Figure 3(A)). Total SBN was slightly increased in male, but not female, flies (Figure 3(B)). Importantly, ABL was not different between young and old flies of either sex (Figure 3(C)), indicating that the quality of sleep did not deteriorate with age in HFD. These results demonstrated that high-nutrient content suppressed the aging-induced deterioration of sleep.

### Long-term exposure to HSD did not suppress aging-induced sleep fragmentation

**Figure 4.**
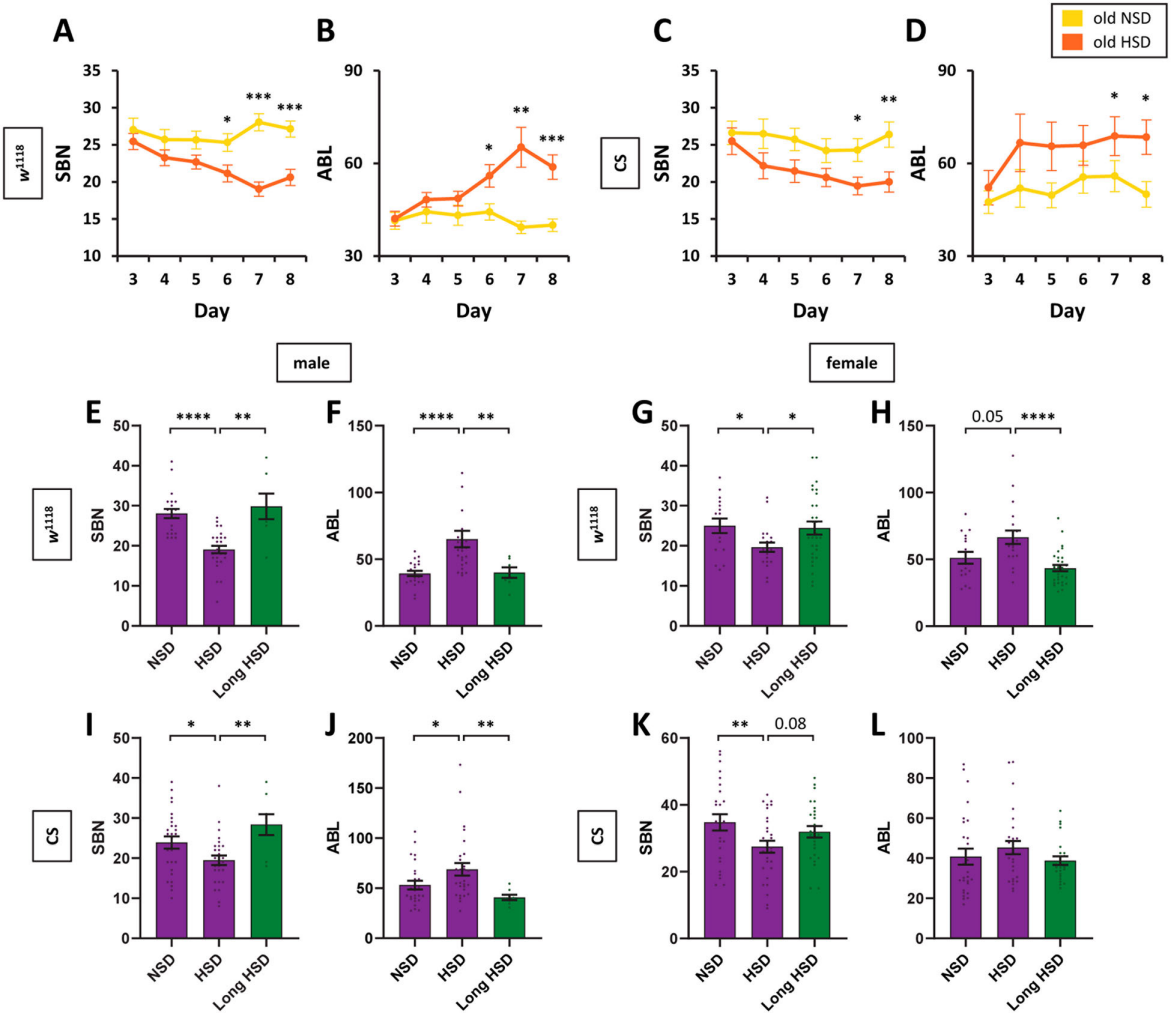
Short-term, but not long-term, exposure to HSD suppressed aging-induced sleep fragmentation. (A, C) SBN and (B, D) ABL of *w*^1118^ and Canton-S old male flies in either NSD or HSD condition were plotted against days. Values indicate mean ± SEM (*n* = 21–32). (E–L) Flies were aged either in NSD and transferred to HSD (NSD, HSD) or kept in HSD from eclosion (long HSD). Sleep analysis of 30-day-old flies fed NSD or HSD (short-term and long-term) was performed. (E, G, I, and K) Number of sleep bouts and (F, H, J, and L) ABL of individual flies were obtained. Bars indicate mean ± SEM (*n* = 9–31). Statistical significance was assessed by independent *t*-test or Mann–Whitney *U* test: *p < 0.05, **p < 0.01, ****p < 0.0001.

Next, we investigated how long-term exposure to HSD would affect aging-induced sleep fragmentation in flies. We compared SBN (Figure 4(A)) and ABL (Figure 4(B)) between young and old *w*^1118^ male flies from day 3 of HSD exposure until day 8. Old flies in HSD tended to have fewer sleep bouts from day 3 until day 5; after day 6, old flies had significantly reduced SBN. The HSD effect on SBN plateaued after 7 days of exposure (Figure 4(A)). For the HSD condition, ABL tended to increase until it reached its peak at day 7. We observed a similar, but less pronounced, pattern of sleep quality improvement for wild-type Canton-S flies fed an HSD (Figure 4(C,D)). Collectively, HSD improved sleep quality in an acute manner, but the effect became saturated at a certain level.

**Figure 5.**
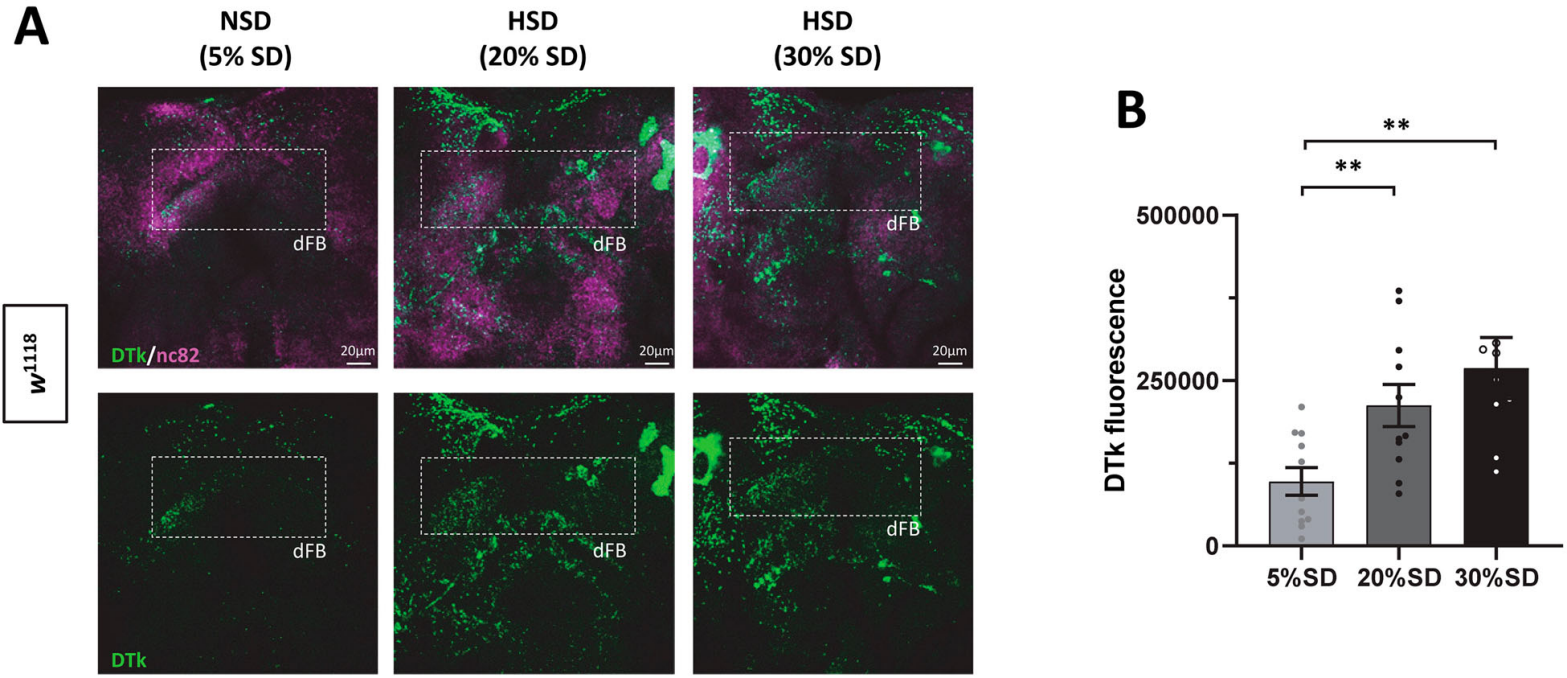
DTk signal was upregulated in the dFB with increasing dietary sucrose. Old *w*^1118^ flies were maintained in NSD or HSD (20% or 30% sucrose) in the incubator with a 12L:12D cycle at 25°C. (A) On day 7, brains were dissected at zeitgeber time 2 (ZT2, ZT0 is light on time) and stained with anti-DTk (green) and anti-nc82 (magenta) antibodies. Dashed box marks DTk positive neurons in dFB. (B) DTk positive signals were quantified using Image J software. Bars indicate mean ± SEM (*n* = 9–14). Statistical significance was assessed by independent *t*-test or Mann–Whitney test: **p < 0.01.

Long-term exposure to HSD could induce numerous physiological changes in *Drosophila* including obesity (Buescher et al. 2013), insulin resistance (Pasco and Leopold 2012), activated immune response (Yu et al. 2018), disrupted ovarian function (Brookheart et al. 2017), and shortened lifespan (Na et al. 2013). In addition, a previous study showed that being kept in a high-calorie diet, which differed from that of the present study, immediately after eclosion accelerated age-dependent sleep fragmentation (Yamazaki et al. 2012). Thus, we examined sleep quality of old *w*^1118^ and Canton-S flies raised in HSD from eclosion until sleep analysis. We compared their sleep quality to that of flies raised in NSD or short-term exposed to HSD (Figure 4(E–L)). Flies in all three different diet conditions were the same ages. Whereas short-term exposure to HSD reduced SBN (Figure 4(E,G,I, and K)) and increased ABL (Figure 4(F,H,J, and L)) in both male and female flies, long-term exposure to HSD for up to 35 days did not greatly alter SBN and ABL compared with NSD. These results were observed in both *w*^1118^ and Canton-S flies, although the difference in ABL for Canton S female flies was not statistically significant. Collectively, these results indicate that long-term exposure to HSD did not have beneficial effects on aging-induced sleep fragmentation (Figure 5).

### Neuropeptide DTk expression was increased in the fan-shaped body of *Drosophila*

Previously, we reported that flies raised in HSD showed the upregulation of Drosophila Tachykinin (DTk) in multiple brain areas (Lee et al. 2021). Thus, we examined whether DTk expression was upregulated in the sleep-regulating center of the *Drosophila* brain. DTk was measured by immunostaining in old flies raised in NSD and HSD conditions. DTk staining was increased in the dorsal fan-shaped body (dFB), which controls sleep. These results suggest that upregulated DTk, a homolog of the mammalian substance P, might increase the sleep quality in HSD by modulating dFB neuronal activity.

## Discussion

Because poor sleep quality puts people at risk of developing serious medical conditions, it is critical to develop an understanding of the many factors that influence sleep. Aging, an inevitable life process, is known to impair sleep quality and thus threaten human health. Emerging evidence suggests that diet influences many aspects of sleep as well (St-Onge et al. 2016). Here, we investigated whether high-nutrient food can suppress aging-induced sleep fragmentation using *Drosophila melanogaster* as a tractable model system. Our results showed that short-term exposure to high-nutrient food suppressed aging-induced sleep fragmentation in flies.

Studies reporting how nutrient content affects sleep are scarce (reviewed in Peuhkuri et al. 2012).

In humans, high-fat/low-carbohydrate morning meal makes subjects more sleepy (Wells et al. 1997), but high-protein diet increases post-prandial alertness (Boelsma et al. 2010). For nighttime sleep, high-carbohydrate/low-fat diet reduces NREM sleep and increases REM sleep (Phillips et al. 1975; Porter and Horne 1981). Consistently, low-carbohydrate/high-fat diet increases the proportion of non-REM sleep (Afaghi et al. 2008). Intriguingly, in mice fed with high-caloric food (HCD) for 12 weeks, a non-rapid eye moment (NREM) sleep episode was more likely to be followed by a rapid eye moment (Yaremchuk 2018) sleep episode and less likely to be followed by a waking period; these results strongly indicate sleep is more consolidated in HCD-fed mice (Panagiotou et al. 2018), which is consistent with the result from this study. Collectively, these studies indicate that ingested food may affect sleep in animals including humans and could be used to increase sleep consolidation.

In this study, high-nutrient food, regardless of sugar or fat composition, prevented aging-induced sleep fragmentation in flies. What might be the underlying mechanism for this effect? In our previous study, flies raised in HSD or HFD for a short duration of time upregulated DTk expression in multiple brain areas (Lee et al. 2021). Tk, an evolutionarily conserved neuropeptide, modulates physiology and numerous behaviors. The mammalian Tk family members are substance P (SP), neurokinin A, and neurokinin B. Studies have shown that SP modulates sleep, although the results have been inconsistent. In mice, SP increases sleep or slows wave sleep depending on the local brain area (Zhang et al. 2004; Zielinski et al. 2015). However, another study found that SP administered intracerebroventricularly increases sleep fragmentation (Andersen et al. 2006). These reports suggest that SP modulates sleep differently depending on the region of the brain it is expressed. In mammals, the ventrolateral preoptic nucleus (VLPO) promotes sleep by inhibiting arousal centers in the brain via GABAergic neurotransmission. In *Drosophila*, the dorsal fan-shaped body (dFB) has been compared to the mammalian VLPO (Donlea et al. 2014; Pimentel et al. 2016). Indeed, the activated dFB releases GABA that inhibits octopaminergic arousal neurons in the medial protocerebrum (Crocker et al. 2010). Additionally, the activation of dFB neurons promotes sleep and greatly increases ABL (Donlea et al. 2011). The dFB is densely supplied with varicose processes of dTk which is likely derived from lateral posterior protocerebrum 1 (LPP1), superior median protecerebrum (SMP), and tritocerebrum 1 (TC1) neurons (Nassel et al. 1988; Lundquist et al. 1994; Winther et al. 2003; Kahsai et al. 2010; Nassel et al. 2019; Lee et al. 2021). Intriguingly, in the previous study, we found out that short-term exposure to HSD increased dTk levels in several neuronal groups including LPP1, SMP, and TC1 neurons in the brain (Lee et al. 2021). Consistently, we observed that dTk staining tended to increase with the increase of sucrose concentration. Thus, upregulated dTk in the dFB might increase sleep quality by modulating the activity of sleep control neurons in dFB in HSD conditions.

Downregulation of the insulin/insulin-like growth factor/TOR signaling network rescues sleep fragmentation of aged flies (Metaxakis et al. 2014). In this report, *Drosophila* insulin-like peptide (Dilp) mutant flies did not show aging-dependent sleep fragmentation. In addition, acute treatment of rapamycin, an inhibitor of TOR signaling, ameliorates sleep fragmentation. Thus, the insulin/insulin-like growth factor/TOR network is a potential candidate for mediating the beneficial effect of HSD on sleep fragmentation. Long-term exposure to HCD appears to result in diabetic changes, such as insulin resistance (Musselman et al. 2011; Na et al. 2013; Baek et al. 2019), with downregulated insulin/insulin-like growth factor signaling in flies. But, long-term exposure to HSD did not reduce aging-induced sleep fragmentation in our study. In fact, diabetes and insulin resistance are reported to impair sleep quality and cause sleep disorders (Perez et al. 2018). Thus, we favored the idea that altered insulin/insulin-like growth factor/TOR signaling network might not be the underlying mechanism for HSD’s effect on sleep fragmentation.

Taken together, in this study, we report that sleep quality of aged flies was improved by short-term maintenance on an HSD. Further studies are needed to more deeply understand the molecular mechanisms through which high-nutrient diets improve sleep quality.
